# Iliac vein stenting outcomes in non-thrombotic and thrombotic diseases: A systematic review and meta-analysis

**DOI:** 10.17305/bb.2025.12777

**Published:** 2025-10-15

**Authors:** Mingxuan Li, Shunquan Wang, Jianwen Zhao, Changzhou Li, Yu Yan, Chuang Shi

**Affiliations:** 1 Department of General Surgery, Beijing Daxing District Hospital of Integrated Chinese and Western Medicine, Beijing, China; 2 Department of Cardiothoracic and Vascular Surgery, Beijing Shijingshan Hospital, Beijing, China; 3 Department of Vascular Surgery, Beijing Fengtai You’anmen Hospital, Beijing, China

**Keywords:** Iliac vein, stents, venous thrombosis

## Abstract

Iliac vein stenting (IVS) is an endovascular revascularization procedure for iliac venous outflow obstruction. We aimed to synthesize the efficacy and safety of IVS across iliac vein disease phenotypes and follow-up horizons. Following a pre-registered protocol (PROSPERO CRD42024606701), we systematically searched Embase, Scopus, PubMed, Web of Science, and Cochrane Library on October 5, 2024. Without restricting study design, we included English-language reports with at least ten patients that reported at least one prespecified outcome (or convertible data) and excluded studies with additional core therapies or duplicated cohorts. Diseases were classified as non-thrombotic iliac vein compression syndrome (NIVCS), post-iliac vein thrombotic syndrome (PIVTS), chronic iliac vein obstruction (CIVO, that is, NIVCS or PIVTS), and acute thrombotic iliac vein obstruction (ATIVO, that is, a CIVO patient with acute ipsilateral thrombosis). The primary outcome was cumulative primary patency (CPP); secondary outcomes comprised ulcer healing, edema and pain relief, quality-of-life improvement, revised Venous Clinical Severity Score change, and adverse events. CPPs at prespecified intervals were extracted for each disease category and pooled in separate meta-analyses. Twenty-seven studies (4782 patients) were included; demographic, intraoperative, and outcome data were systematically abstracted. Pooled CPPs were consistently high, particularly for NIVCS, and were lower when thrombotic components were present (PIVTS and ATIVO), while other efficacy outcomes generally improved and serious complications were uncommon. In conclusion, across diverse iliac vein diseases and follow-up periods, IVS demonstrates good efficacy and safety; this unfunded study supports IVS as a prominent treatment option.

## Introduction

In 1957, May and Thurner [1] first reported abnormal obstructive hyperplasia of the iliac vein wall resulting from chronic compression. Cockett and Thomas [2] subsequently termed this condition “iliac vein compression syndrome” based on findings from venography and surgical procedures. This syndrome can lead to chronic venous congestion in the lower limbs, manifesting in a range of clinical symptoms, and is a prevalent cause of chronic venous disease (CVD) [3]. Furthermore, a significant proportion of patients with acute deep vein thrombosis (DVT) in the lower limbs exhibit ipsilateral chronic iliac vein occlusion [4, 5]. To differentiate this condition from IVO without acute thrombosis, we refer to it as acute thrombotic IVO (ATIVO). The residual thrombus in the iliac vein following acute DVT may result in post-thrombotic syndrome (PTS), a specific form of CVD known as post-iliac vein thrombotic syndrome (PIVTS). We categorize non-thrombotic iliac vein compression syndrome (NIVCS), which lacks acute or chronic thrombotic components, and PIVTS collectively as chronic IVO (CIVO).

Typically, revascularization for CIVO patients is considered only when symptoms are pronounced [3]. In the case of ATIVOs, luminal stenosis of at least 50% is recognized as the anatomical indication for revascularization in all patients with IVO [6–8]. Endovascular procedures are the first-line treatment for IVOs [3]. It is widely accepted that percutaneous transluminal angioplasty (PTA) alone is often insufficient for treating IVOs due to the frequent immediate elastic recoil of the treated vein segment, necessitating the use of stenting [3, 9, 10]. Consequently, the patency of stents following iliac vein stenting (IVS) and its associated clinical efficacy have become focal points of research. Recent years have seen an increase in studies reporting on these outcomes; however, the majority of them lack robust classical controls and are predominantly single-arm studies [11–13].

Notably, post-IVS cumulative primary patencies (CPPs) vary significantly according to patient categories. For instance, Kwak et al. [14] reported a 2-year postoperative CPP of 95.5% for ATIVO patients, while Kim et al. [15] reported only 70.5%. Additionally, a range of adverse events, such as back pain, stent thrombosis, and contralateral DVT, have been documented, with varying rates of occurrence. For example, Moini et al. [13] reported a cumulative stent thrombosis rate of 10.2% during a 6-month follow-up for PIVTS patients, whereas no such events were reported by Tang et al. [16] in their 2-year follow-up of CIVO patients. In light of these disparities and the absence of comprehensive reviews, we aimed to gain a thorough understanding of the efficacy and safety outcomes of IVS for different IVO patients across various follow-up periods. To achieve this, we conducted a systematic review and performed meta-analyses focusing on different CPPs as the primary outcomes of interest.

## Materials and methods

### Study protocol

Following the PRISMA framework, this study was registered on the PROSPERO platform (CRD42024606701). All data were sourced from published literature and did not include any individual identifying information; thus, ethical approvals and patient consent forms were not required. The PRISMA 2020 checklist is provided in Table S1.

### Search strategy

A systematic search of the Excerpta Medica Database (Embase), Scopus, PubMed, Web of Science (WOS), and Cochrane Library was conducted on October 5, 2024. The primary search criteria included any literature with titles containing “iliac vein/iliac venous” and “stent/stenting.” The literature search was independently executed by ML, and the search strings utilized in each database are detailed in Table S2.

### Study selection

The post-IVS CPP was established as the primary outcome of interest, with secondary outcomes encompassing postoperative efficacy and safety metrics. Efficacy outcomes included ulcer healing, edema relief, pain relief, quality of life (QoL) improvement, and revised Venous Clinical Severity Score (rVCSS) improvement [17]. Safety outcomes included foreign body sensation, back pain, puncture hematoma, pulmonary embolism (PE), all-cause mortality, stent thrombosis, stent fracture, stent collapse, contralateral DVT, ipsilateral DVT/recurrence, and PTS. All outcomes adhered to their original definitions as reported in the literature and were planned to be recorded as cumulative rates over specified periods. Inclusion criteria for the review required that literature reported at least one outcome of interest in the specified format or provided data for indirect calculation, while meeting the following conditions: 1) publication in English; 2) not solely published in abstract form; 3) no other core treatment modalities aside from IVS were part of the study; 4) a minimum of ten patients in the population series; and 5) no duplication of data with other published literature.

All retrieved literature was imported into Endnote 21 software, followed by duplicate removal and abstract review. Subsequently, the full texts of all literature passing preliminary screening were downloaded and assessed for final inclusion in the study. Two authors (ML and SW) independently executed the study selection, resolving any discrepancies by consensus.

### Data extraction

Following the identification of relevant literature, data regarding the literature, population, IVS procedures, follow-up periods, and each outcome of interest were extracted. All outcome measures were expressed as cumulative percentages with 95% confidence intervals (CIs), calculated by dividing the cumulative number of cases over a specified period by the total number of cases at the start of follow-up. Missing data were excluded from both the numerator and denominator. Data extraction was conducted by two independent authors (ML and JZ), with any discrepancies resolved through consensus.

### Quality assessment

The included literature was evaluated using the Joanna Briggs Institute (JBI) checklist [18] and the Agency for Healthcare Research and Quality’s (AHRQ) cross-sectional study quality evaluation items [19] concurrently. The JBI quality assessment tool for prevalence research consists of nine items assessing the overall quality of studies based on sampling methods, research subjects, data collection, and analysis techniques; each item received a score of 1 point for a “yes” response and 0 points for “no,” “not clear,” or “not applicable.” The AHRQ evaluation items comprise 11 domains, with “yes” scored as 1 point and “no” or “not clear” scored as 0 points. Included literature was categorized as having “low” (0–3 points), “medium” (4–7 points), or “high” (8–11 points) methodological quality, adopting the lower quality classification from the two assessment systems. Quality assessment was performed by two independent authors (ML and CL), with any disagreements resolved by selecting the lower score.

### Statistical analysis

Statistical analyses were conducted using Stata (Stata Corp., College Station, TX, USA) version 16.0. Meta-analyses of all outcome measures utilized the Metaprop command [20] with the Freeman–Tukey (F-T) double arcsine transformation [21] to derive pooled effect sizes (ESs) and 95% CIs. Both fixed and random effects models were applied in the analyses [22]. The basic meta-analysis command employed was: metaprop e n, random ftt cimethod (exact). In addition to textual descriptions, the pooled analysis results of the outcomes of interest are illustrated as forest plots. Only results with a *P* value less than 0.05 were considered statistically significant. All pooled analyses were performed independently by ML.

**Figure 1.**
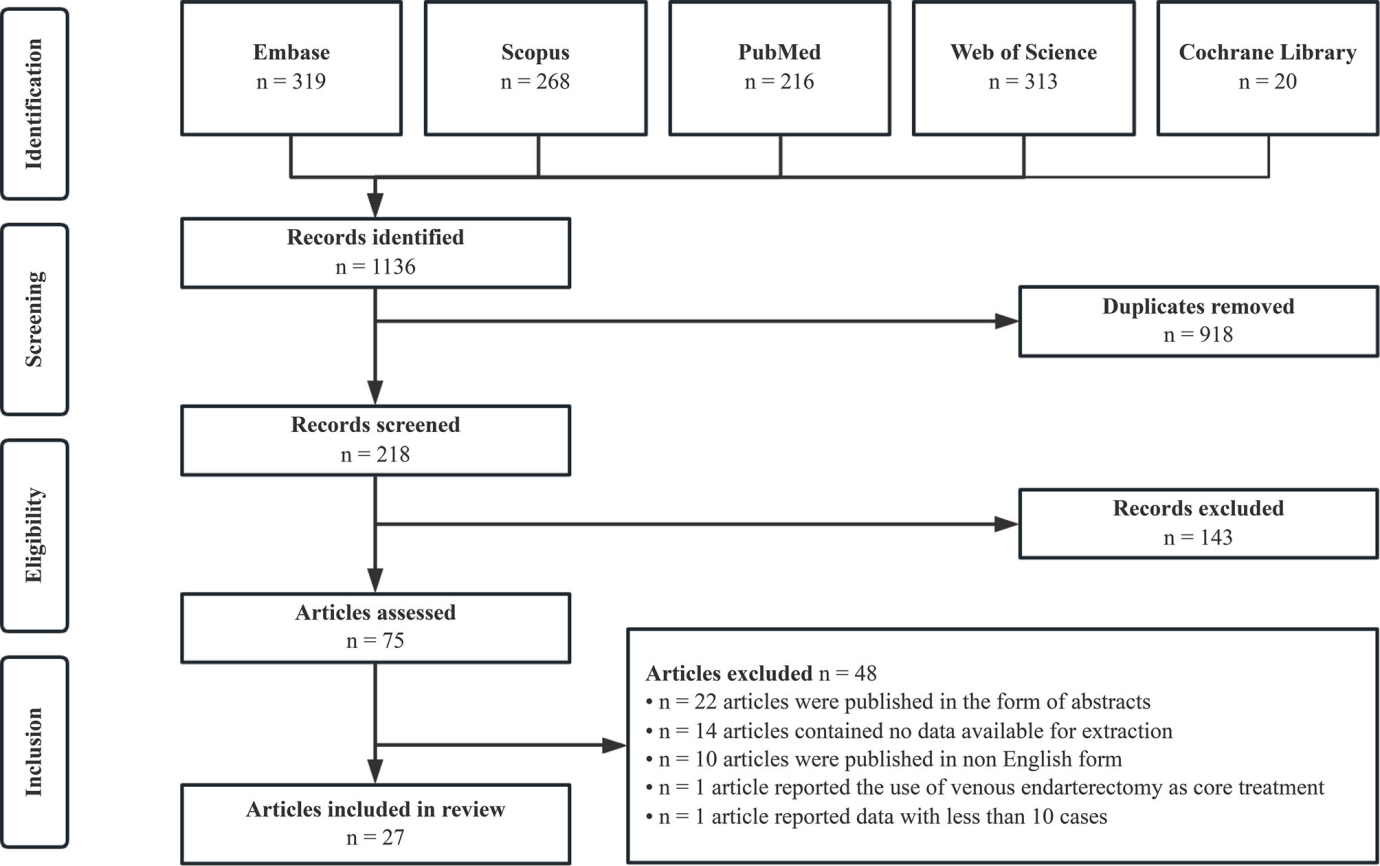
**PRISMA flowchart**.

**Table 1:** Characteristics and assessment results of literatures

**References**	**JBI score**	**AHRQ score**	**Quality level**	**Publication year**	**Study type**	**Country**	**Enrolled year**	**Enrolled population**	**Population amount**
Xue [27]	7	8	M	2014	RO	China	2006–2011	ATIVO	61
Raju [28]	8	8	H	2002	RO	USA	1997–2000	CIVO	292
Jiang [29]	7	9	M	2024	RO	China	2020–2022	NIVCS	55
Jiang [29]	7	9	M	2024	RO	China	2020–2022	PIVTS	28
Le [11]	9	8	H	2018	RO	Korea	2004–2017	NIVCS	111
Ming [12]	9	8	H	2017	RO	China	2011–2015	ATIVO	116
Lim [30]	7	7	M	2020	RO	Singapore	2014–2019	CIVO	87
Kim [31]	9	9	H	2020	RO	Korea	2004–2018	ATIVO	130
Satwah [32]	9	7	M	2021	RO	USA	2015–2019	CIVO	1104
Alsheekh [33]	8	8	H	2017	RO	USA	2012–2014	NIVCS	623
Ye [34]	8	8	H	2012	RO	China	2000–2012	NIVCS	205
Jeon [10]	7	8	M	2010	RO	Korea	1999–2007	ATIVO	30
Jiang [35]	8	8	H	2019	RO	China	2014–2016	ATIVO	46
Raju [36]	6	6	M	2014	RO	USA	NE	CIVO	NE
Snow [37]	9	7	M	2023	RO	USA	2014–2021	CIVO	627
Dasari [38]	7	8	M	2017	RO	USA	2007–2014	ATIVO (pregnancy)	11
Kim [15]	8	8	H	2022	RO	Korea	2001–2018	ATIVO	44
Abdul-Haqq [39]	8	8	H	2017	RC	UK	2003–2015	NIVCS	NE
Abdul-Haqq [39]	8	8	H	2017	RC	UK	2003–2015	PIVTS	NE
Rizvi [40]	8	8	H	2018	RO	USA	2013–2014	NIVCS	210
Kwak [14]	7	7	M	2005	RO	Korea	2000–2004	ATIVO	22
Moini [13]	9	8	H	2019	RC	Iran	2015–2017	NIVCS	88
Moini [13]	9	8	H	2019	RC	Iran	2015–2017	PIVTS	76
Lichtenberg [41]	8	8	H	2021	PC	Germany	2016–2017	NIVCS	29
Lichtenberg [41]	8	8	H	2021	PC	Germany	2016–2017	PIVTS	50
Foegh [42]	7	6	M	2022	RO	Denmark	NE	ATIVO	45
Tang [16]	7	7	M	2022	PC	Singapore	2018–2019	CIVO	60
Hügel [43]	9	8	H	2022	PC	Switzerland	2008–2020	CIVO	108
Robertson [44]	8	8	H	2022	RO	USA	2016–2021	NIVCS	41
Robertson [44]	8	8	H	2022	RO	USA	2016–2021	PIVTS	38
Robertson [44]	8	8	H	2022	RO	USA	2016–2021	ATIVO	29
Cooke [45]	9	8	H	2022	PO	USA	2011–2021	CIVO	376
Taha [46]	7	7	M	2020	PO	Egypt	2016–2019	CIVO	40

Abbreviations: JBI: Joanna Briggs Institute; AHRQ: Agency for Health Care Research and Quality; M: Medium; H: High; RO: Retrospective observation; RC: Retrospective control; PC: Prospective control; PO: Prospective observation; NE: Not extracted; ATIVO: Acute thrombotic iliac vein occlusion; CIVO: Chronic iliac vein occlusion; NIVCS: Non-thrombotic iliac vein compression syndrome; PIVTS: Post-iliac vein thrombotic syndrome.

**Table 2:** Data on CPP

**References**	**Enrolled population**	**Mean follow-up period**	**CPP-6mo, %**	**CPP-1y, %**	**CPP-2y, %**	**CPP-3y, %**	**CPP-4y, %**	**CPP-5y, %**
Xue [27]	ATIVO	31mo	95.1	91.8	90.2	88.5	NE	85.2
Raju [28]	CIVO	2y	NE	NE	71.0	NE	NE	NE
Jiang [29]	NIVCS	3y	94.5	94.5	94.5	94.5	NE	NE
Jiang [29]	PIVTS	3y	88.5	85.4	85.4	85.4	NE	NE
Le [11]	NIVCS	3y	NE	NE	NE	NE	NE	NE
Ming [12]	ATIVO	NE	NE	NE	NE	NE	NE	NE
Lim [30]	CIVO	NE	95.7	92.8	NE	NE	NE	NE
Kim [31]	ATIVO	14mo	NE	NE	NE	NE	NE	NE
Satwah [32]	CIVO	NE	NE	NE	NE	NE	NE	NE
Alsheekh [33]	NIVCS	1y	NE	NE	NE	NE	NE	NE
Ye [34]	NIVCS	4y	NE	NE	NE	NE	98.7	NE
Jeon [10]	ATIVO	1y	NE	83.3	NE	NE	NE	NE
Jiang [35]	ATIVO	2y	97.8	95.7	91.1	NE	NE	NE
Raju [36]	CIVO	2y	NE	NE	69.1	NE	NE	NE
Snow [37]	CIVO	NE	NE	NE	NE	NE	NE	NE
Dasari [38]	ATIVO (pregnancy)	63mo	NE	NE	NE	NE	NE	90.9
Kim [15]	ATIVO	25mo	NE	70.5	70.5	NE	NE	NE
Abdul-Haqq [39]	NIVCS	20mo	NE	NE	NE	97.2	NE	NE
Abdul-Haqq [39]	PIVTS	20mo	NE	NE	NE	73.7	NE	NE
Rizvi [40]	NIVCS	499d	98.7	98.3	97.9	NE	NE	NE
Kwak [14]	ATIVO	21mo	NE	95.5	95.5	NE	NE	NE
Moini [13]	NIVCS	6mo	98.8	NE	NE	NE	NE	NE
Moini [13]	PIVTS	6mo	88.2	NE	NE	NE	NE	NE
Lichtenberg [41]	NIVCS	24mo	NE	NE	95.5	NE	NE	NE
Lichtenberg [41]	PIVTS	24mo	NE	NE	96.0	NE	NE	NE
Foegh [42]	ATIVO	13y	NE	NE	NE	NE	NE	> 94.0^*^
Tang [16]	CIVO	29mo	NE	92.4	87.1	NE	NE	NE
Hügel [43]	CIVO	41mo	NE	90.7	84.3	83.3	82.4	82.4
Robertson [44]	NIVCS	6mo	97.6	NE	NE	NE	NE	NE
Robertson [44]	PIVTS	6mo	84.2	NE	NE	NE	NE	NE
Robertson [44]	ATIVO	6mo	86.2	NE	NE	NE	NE	NE
Cooke [45]	CIVO	6mo	NE	NE	NE	NE	NE	NE
Taha [46]	CIVO	17mo	80.0	76.0	NE	NE	NE	NE

*: Data reported in the literature is a value that extend beyond post-operative 5 years. Abbreviations: ATIVO: Acute thrombotic iliac vein occlusion; CIVO: Chronic iliac vein occlusion; NIVCS: Non-thrombotic iliac vein compression syndrome; PIVTS: Post-iliac vein thrombotic syndrome; mo: Months; y: Year(s); d: Days; NE: Not extracted; CPP: Cumulative primary patency.

**Table 3:** Meta-analyses of various CPPs and certainties of evidences

**Outcome**	**Included literatures initially**	**Heterogeneity of initial REM (*I*^2^ and *P* for *Q* test)**	**Omitted literature**	**Heterogeneity of new REM (*I*^2^ and *P* for *Q* test)**	**Population size**	**ES obtained from new REM**	**ES obtained from final FEM**	**Sensitivity analysis result**	**Publication bias and p for Egger’s test**	**Certainty of evidence^a^**	**Alteration to initial rating**
6mo CPP of NIVCS	[13, 29, 40, 44]	56.742%, 0.074	[29]	12.651%, 0.318	339	0.995 (95% CI, 0.981–1.000)	0.996 (95% CI, 0.984–1.000)	Stable	Low, 0.084	Low	0
2y CPP of NIVCS	[29, 40, 41]	39.798%, 0.190	NA	NA	294	0.977 (95% CI, 0.940–0.998)	0.984 (95% CI, 0.965–0.996)	Unstable	Low, 0.392	Low	0
6mo CPP of PIVTS	[13, 29, 44]	< 0.001%, 0.800	NA	NA	142	0.875 (95% CI, 0.813–0.927)	0.875 (95% CI, 0.813–0.927)	Stable	Low, 0.813	Low	0
2y CPP of CIVO	[16, 28, 36, 43]	71.088%, 0.016	[43]	< 0.001%, 0.549	569	0.710 (95% CI, 0.672–0.746)	0.710 (95% CI, 0.672–0.746)	Stable	Low, 0.418	Low	0
6mo CPP of ATIVO	[27, 35, 44]	43.981%, 0.168	NA	NA	136	0.944 (95% CI, 0.876–0.989)	0.948 (95% CI, 0.901–0.982)	Stable	Low, 0.435	Low	0
1y CPP of ATIVO	[14, 15, 27, 10, 35]	71.722%, 0.007	[15]	7.976%, 0.353	159	0.924 (95% CI, 0.871–0.965)	0.924 (95% CI, 0.875–0.963)	Stable	Low, 0.828	Low	0
2y CPP of ATIVO	[14, 15, 27, 35]	69.809%, 0.019	[15]	< 0.001%, 0.831	129	0.917 (95% CI, 0.860–0.962)	0.917 (95% CI, 0.860–0.962)	Stable	High, 0.012	Very low	-1^b^

^a^: High certainty: We are very confident that the true effect lies close to that of the estimate of the effect; moderate certainty: We are moderately confident in the effect estimate; low certainty: Our confidence in the effect estimate is limited; very low certainty: We have very little confidence in the effect estimate. ^b^: The reason for lowering the rating was a a suspect of publication bias. Abbreviations: CPP: Cumulative primary patency; NIVCS: Non-thrombotic iliac vein compression syndrome; PIVTS: Post-iliac vein thrombotic syndrome; CIVO: Chronic iliac vein occlusion; ATIVO: Acute thrombotic iliac vein occlusion; REM: Random effects model; NA: Not applicable; ES: Effect size; CI: Confidence interval; FEM: Fixed effects model.

### Heterogeneity assessment

The initial analysis employed a random effects model. Heterogeneity was assessed and reported as a percentage using the *I*^2^ index [23] and as a *P* value utilizing the Cochrane *Q* test [24]. If Heterogeneity was classified as high if the *I*^2^ statistic was ≥ 50% or the *P* value was ≤0.10; otherwise, it was deemed low. In cases of high heterogeneity, the literature with the lowest weight in the model was excluded, and the calculation was repeated. If high heterogeneity persisted, the previously excluded literature was re-included, and the literature with the second lowest weight was removed, continuing this process until a model with low heterogeneity was achieved. Only random effects models that included at least three studies with low heterogeneity were initially accepted. The outcomes of the literature in this model were then re-pooled and analyzed using a fixed effects model to derive the final adopted ES. Heterogeneity assessment was conducted independently by YY.

### Sensitivity analysis

Following the establishment of the final adopted model, sensitivity analysis was performed by omitting each included study one at a time to examine the model’s stability. The natural logarithm (ln) conversion was applied to all new ES values derived from these recalculations. If an ln(new SE) value significantly deviated from the ES of the previous final model or fell outside its 95% CI, the model was considered unstable. Sensitivity analysis was conducted independently by YY.

### Publication bias assessment

Publication bias for each final adopted model was evaluated using Egger’s test [25]. A *P* value less than 0.05 indicated a high likelihood of bias. Additionally, funnel plots were generated; an asymmetric plot reflecting ESs indicated potential bias [26]. The publication bias assessment was performed independently by CS.

### Evidence quality grade assessment

Following the completion of the meta-analyses, we employed the Grading of Recommendations Assessment, Development and Evaluation (GRADE) system to assess the quality of evidence and formulate recommendations. Each result was classified as high, moderate, low, or very low. Given that all included studies were retrospective, the initial rating was set to low, with adjustments made as warranted. The assessment was conducted independently by CS.

## Results

### Characteristics of the literature

We initially identified 1136 articles through searches of five academic databases, ultimately evaluating 218 after removing duplicates. Seventy-five articles were retained following a title and abstract review. A thorough review of the full texts led to the inclusion of 27 articles in this study [10, 11–16, 27–46]. The PRISMA flowchart detailing the study selection process is presented in Figure 1.

The publication years of the included articles ranged from 2002 to 2024, with the majority (74.1%) being retrospective observational studies. Our assessment indicated that 55.6% of the articles were of high quality, with no articles rated as low quality. The sample populations originated predominantly from the United States (29.6%), China (18.5%), and various other countries. Some articles reported data from multiple populations, leading to the extraction of 33 case series, which were categorized into the NIVCS group (9 series), PIVTS group (5), CIVO group (9), and ATIVO group (10). The largest series comprised 1104 patients (CIVOs) [32], while the smallest included 11 patients (pregnant ATIVOs) [38]. The characteristics and assessment outcomes of the literature are summarized in Table 1.

### Preoperative and intraoperative data of patients

With the exception of literature reporting solely on ATIVOs with an average age of 28 [38], the average age of included patients ranged from 41.9 years (ATIVOs) to 72.0 years (NIVCSs) [39, 40]. The proportion of females varied from 25.0% (NIVCSs) to 86.7% (ATIVOs) [13, 42]. Compared to the contralateral limbs, the left lower limbs exhibited higher prevalence rates of disease. In terms of the Clinical, Etiological, Anatomical, and Pathophysiological (CEAP) classification [47], the majority of patients fell within the C3 to C4 categories. Extracted data indicated that the diameter of stents used was predominantly less than 20 mm, and the total length of unilateral stents post-implantation was mainly under 100 mm. The preoperative and intraoperative data of patients are summarized in Table S3.

### Summary of CPP data across different follow-up periods

Reported follow-up durations varied from 6 months to 13 years [13, 42, 44, 45]. Based on the available data, the CPPs at 6 months, 1 year, 2 years, 3 years, 4 years, and 5 years for various patient series were extracted. Except for one study on CIVOs, the CPPs at 6 months and 1 year consistently exceeded 80% [46]. The CPP data at different follow-up intervals are summarized in Table 2.

**Figure 2.**
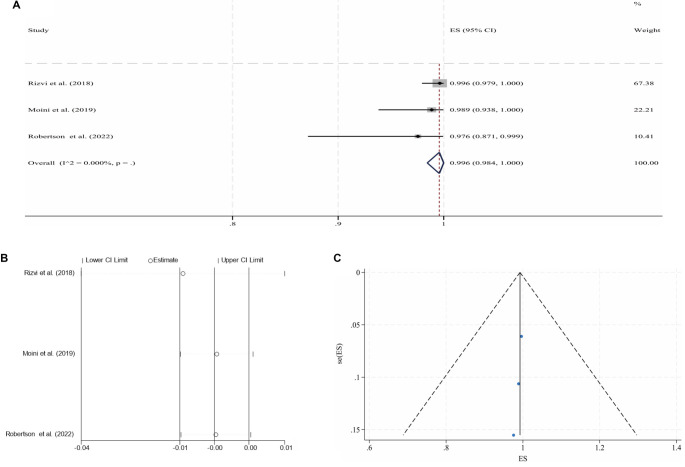
**Pooled analysis for the 6-month CPP of NIVCSs (fixed-effect model).** (A) Forest plot of individual studies and the overall estimate; (B) Leave-one-out sensitivity analysis (each study omitted in turn); (C) Funnel plot with pseudo 95% confidence limits. Abbreviations: NIVCS: Non-thrombotic iliac vein compression syndrome; CPP: Cumulative primary patency; ES: Effect size; CI: Confidence interval.

**Figure 3.**
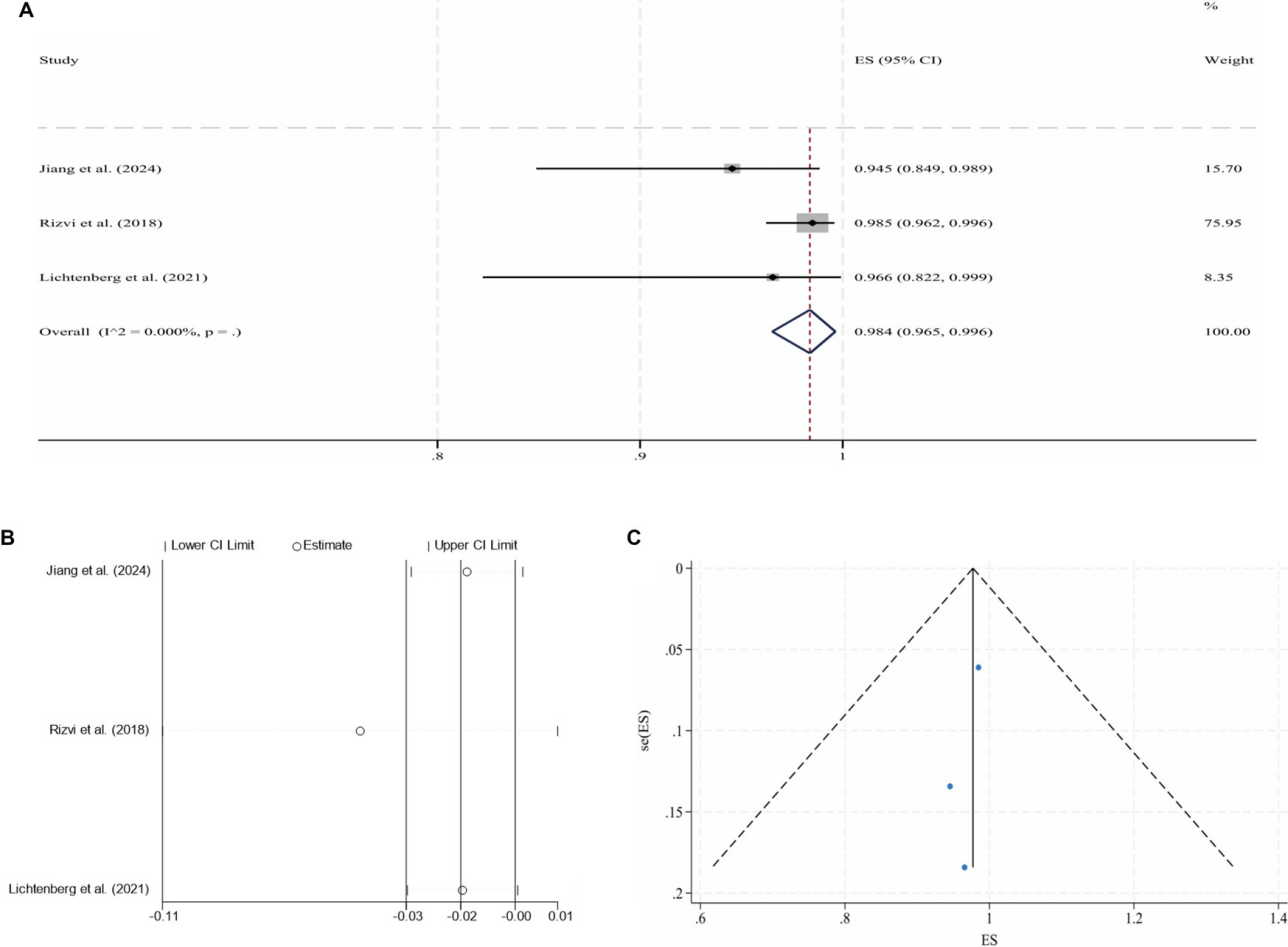
**Pooled analysis for the 2-year CPP of NIVCSs (fixed-effect model).** (A) Forest plot of individual studies and the overall estimate; (B) Leave-one-out sensitivity analysis (each study omitted in turn); (C) Funnel plot with pseudo 95% confidence limits. Abbreviations: NIVCS: Non-thrombotic iliac vein compression syndrome; CPP: Cumulative primary patency; ES: Effect size; CI: Confidence interval.

### Meta-analyses of various CPPs and certainties of evidences

**Figure 4.**
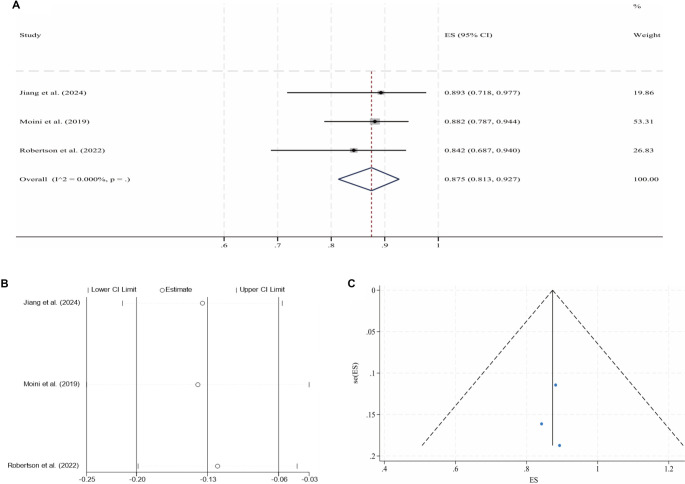
**Pooled analysis for the 6-month CPP of PIVTSs (fixed-effect model).** (A) Forest plot of individual studies and the overall estimate; (B) Leave-one-out sensitivity analysis (each study omitted in turn); (C) Funnel plot with pseudo 95% confidence limits. Abbreviations: PIVTS: Post-iliac vein thrombotic syndrome; CPP: Cumulative primary patency; ES: Effect size; CI: Confidence interval.

**Figure 5.**
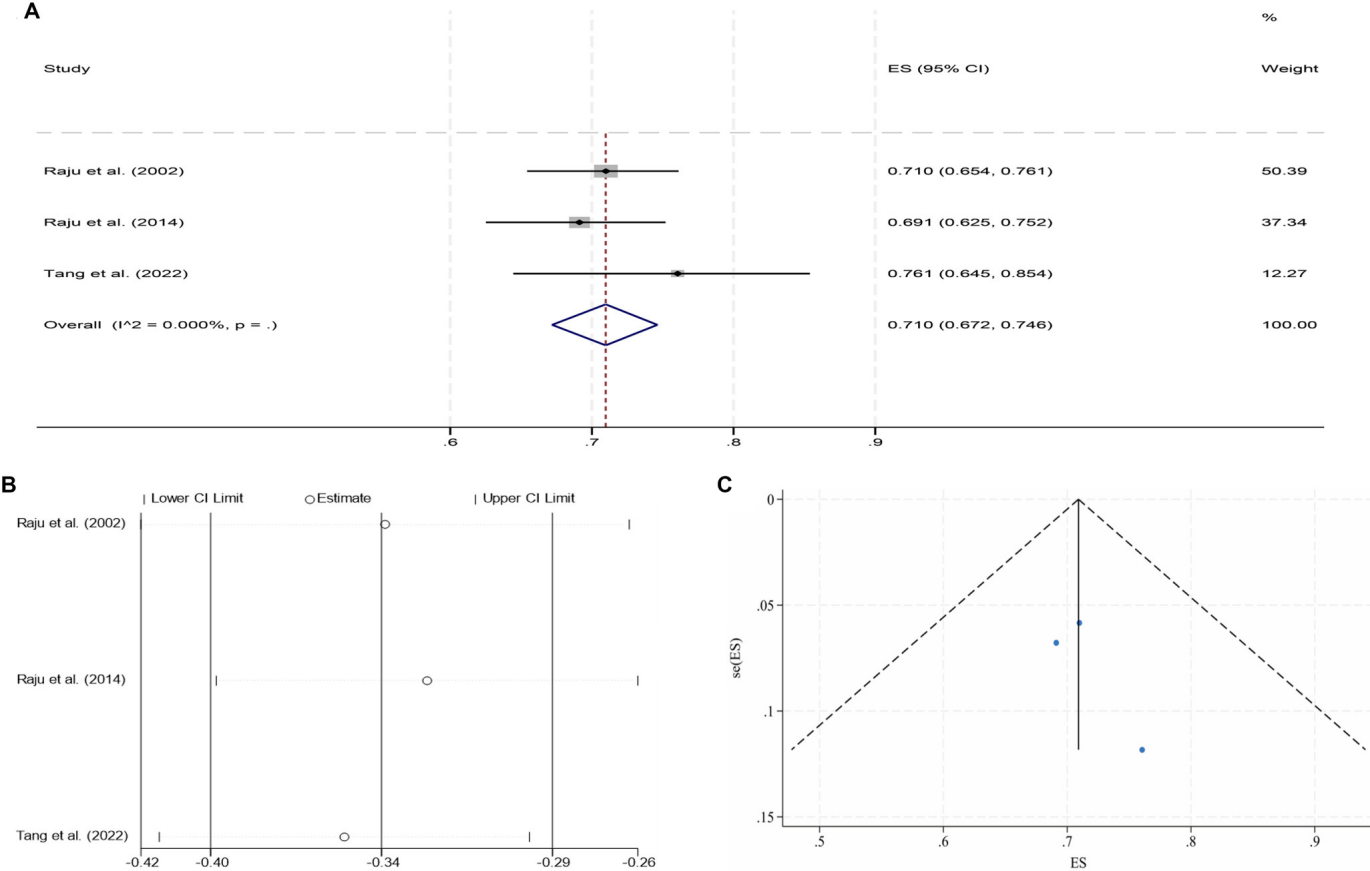
**Pooled analysis for the 2-year CPP of CIVOs (fixed-effect model).** (A) Forest plot of individual studies and the overall estimate; (B) Leave-one-out sensitivity analysis (each study omitted in turn); (C) Funnel plot with pseudo 95% confidence limits. Abbreviations: ES: Effect size; CI: Confidence interval; CPP: Cumulative primary patency; CIVO: Chronic iliac vein obstruction.

**Figure 6.**
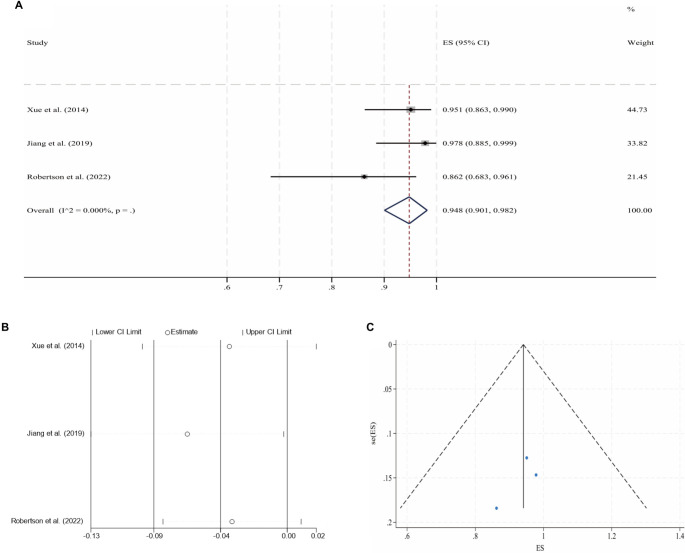
**Pooled analysis for the 6-month CPP of ATIVOs (fixed-effect model).** (A) Forest plot of individual studies and the overall estimate; (B) Leave-one-out sensitivity analysis (each study omitted in turn); (C) Funnel plot with pseudo 95% confidence limits. Abbreviations: ATIVO: Acute thrombotic iliac vein obstruction; CPP: Cumulative primary patency; ES: Effect size; CI: Confidence interval.

**Figure 7.**
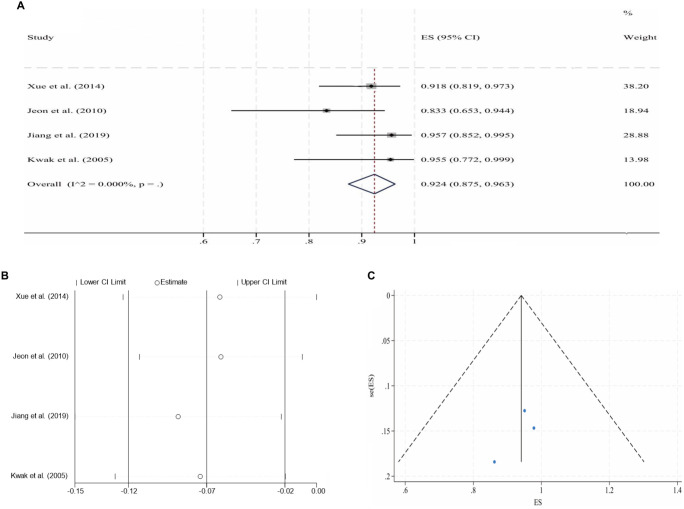
**Pooled analysis for the 1-year CPP of ATIVOs (fixed-effect model).** (A) Forest plot of individual studies and the overall estimate; (B) Leave-one-out sensitivity analysis (each study omitted in turn); (C) Funnel plot with pseudo 95% confidence limits. Abbreviations: ATIVO: Acute thrombotic iliac vein obstruction; CPP: Cumulative primary patency; ES: Effect size; CI: Confidence interval.

**Figure 8.**
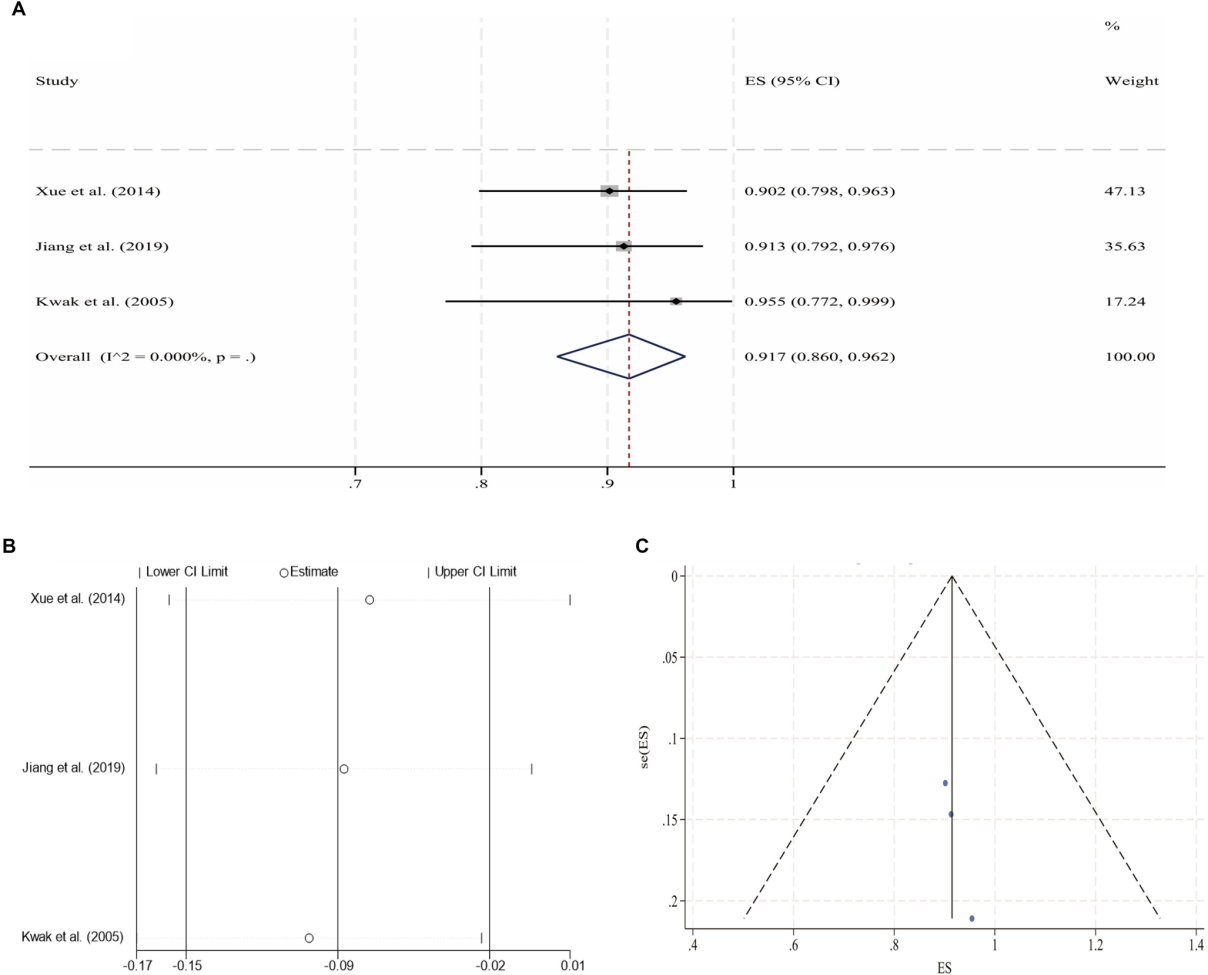
**Pooled analysis for the 2-year CPP of ATIVOs (fixed-effect model).** (A) Forest plot of individual studies and the overall estimate; (B) Leave-one-out sensitivity analysis (each study omitted in turn); (C) Funnel plot with pseudo 95% confidence limits. Abbreviations: ATIVO: Acute thrombotic iliac vein obstruction; CPP: Cumulative primary patency; ES: Effect size; CI: Confidence interval.

Following IVS, the 6-month and 2-year CPPs for NIVCSs, the 6-month CPPs for PIVTSs, and the 1-year and 2-year CPPs for CIVOs, as well as the 6-month, 1-year, and 2-year CPPs for ATIVOs, were pooled for meta-analysis due to the availability of sufficient literature data. Due to universally high heterogeneity observed in the random effects models when pooling results from the remaining three studies, the meta-analysis results for the 1-year CPP of CIVOs were not included [16, 30, 43, 46]. Other pooled analyses yielded models with low heterogeneity, although the model for the 2-year CPP of ATIVOs exhibited significant publication bias (*P* ═ 0.012, Egger’s test). The processes of meta-analyses and the certainties of the obtained evidence are presented in Table 3. The forest plots, sensitivity analyses, and funnel plots for each adopted model are illustrated in Figures 2–8.

### Efficacy outcomes of IVS beyond CPP

Although limited data were available regarding efficacy outcomes beyond CPP, results were generally favorable (Table S4). Moini et al. [13] reported cumulative complete ulcer healing rates of 31.0% for PIVTSs and 42.4% for NIVCSs at 6 months post-IVS. Additionally, three other studies documented a post-IVS cumulative rate of 100% for CIVOs at 17, 24, and 29 months [16, 41, 46]. The cumulative edema relief rate for CIVOs at 6 months ranged from 80.0% to 100% [13, 45]. Ye et al. [34] indicated that the cumulative rate for NIVCSs reached 89.1% by the fourth year. Moini et al. [13] were the only authors to report specific pain relief rates, which were cumulative rates of 98.7% for PIVTSs and 100% for NIVCSs at the 6-month mark. Raju et al. [28] reported a 2-year cumulative QoL improvement rate of 46.6% for CIVOs. It was noted that rVCSS scores decreased by an average of 4.72 and 7.77 for NIVCSs and PIVTSs, respectively, at the 6th postoperative month [13]. Lim et al. [30] did not specify a follow-up period but noted an average decrease of 5.75 in rVCSS scores for CIVOs. Although specific rates or values were not disclosed, several studies indicated statistically significant changes (*P* < 0.05) in efficacy variables for various CIVOs following IVS [16, 28, 30, 34, 41, 46].

### Safety outcomes of IVS

The included studies reported various adverse events following IVS, which were challenging to classify and were primarily postoperative rather than intraoperative (Table S5). Representative events and their cumulative rates were extracted. Notable discrepancies in the incidence of back pain were observed across studies (1.6% for ATIVOs compared to 66.0% for CIVOs), although all cases exhibited a tendency for self-resolution [27, 37]. Serious adverse events were infrequent, with all-cause mortality rates ranging from 0% to 4.5% (up to 21 months of follow-up) [14, 41]. Reported studies consistently indicated an incidence of PE at 0% [16, 29, 35, 43, 46]. Among stent-related adverse events, stent collapse and stent thrombosis were slightly more prevalent, with incidences reported between 3.3% and 11.3% and 0% and 10.2%, respectively [10, 13, 33, 42]. DVT events were occasionally reported, with incidence rates ranging from 0% to 8.5% on the ipsilateral side and 0% to 11.4% on the contralateral side [15, 16, 31, 41]. Furthermore, the incidence of PTS in ATIVOs after IVS was between 2.2% and 21.6% [12, 35].

## Discussion

This study systematically reviewed and analyzed outcomes across populations at different follow-up intervals following IVS. The results indicate that IVS demonstrates satisfactory efficacy and safety. This discussion focuses on the generalizability of findings across diverse populations, healthcare systems, and stenting techniques.

The prevalence of IVO is notably high. Kibbe et al. [48] reported significant iliac vein compression in as many as 24% of an asymptomatic population. Diagnosis rates for IVO have been reported at 15% among patients with CVD and 30% among those with DVT [4, 5, 34]. Chen et al. [49] highlighted that iliac vein stenosis exceeding 50% can increase the risk of DVT by approximately tenfold. As clinicians gain a deeper understanding of various IVOs, the accuracy of diagnosis is improving.

The etiology of most CIVO lesions can be categorized into two types: external compression and residual thrombus within the lumen [1, 50, 51]. IVS is a promising treatment method for these conditions. Notably, the post-IVS CPPs for the aforementioned conditions differ significantly [29, 40, 44]. Furthermore, Kim et al.’s study [15] suggested that fresh thrombosis at the CIVO lesion tends to reduce postoperative CPP. Consequently, we categorized IVO into three distinct types—NIVCS, PIVTS, and ATIVO—and analyzed their postoperative CPPs to mitigate bias due to heterogeneity.

Our review found that most IVO lesions in the included studies were located on the left side, with some studies reporting exclusively left-sided cases [15, 31]. This aligns with findings from other studies [1, 4, 5]. We propose that congenital anatomical factors render the left iliac vein more susceptible to external compression compared to the contralateral vein. The clinical characteristics of patients with different types of IVO vary considerably. CIVO, a common contributor to CVD, can result in mild varicose veins or severe, non-healing skin ulcers. Widely used evaluation methods based on clinical manifestations include the CEAP classification, the Villalta scale, and the rVCSS [17, 47, 52]. Venous claudication, typically described as heaviness and pain during exertion that subsides with rest, remains a subjective and poorly validated symptom [53]. Although not included in formal scoring systems, it may influence clinical decision-making [3]. Conversely, ATIVO patients primarily present with acute symptoms, and severity assessment focuses on the burden and location of thrombus to predict the risk of PE [54].

For ATIVO patients, the indication for IVS generally includes imaging-confirmed iliac vein diameter or area stenosis of at least 50% and removal of the majority of fresh thrombus at the site of stenosis, even in the absence of prior CIVO clinical manifestations [12, 15, 27, 35]. IVO is widely recognized as a risk factor for DVT recurrence [55–57]; thus, we advocate for the combined use of IVS and thrombectomy. Conversely, for CIVO patients, even with anatomical characteristics indicative of stenosis (≥50%), if chronic clinical manifestations are mild or absent, stenting is typically deemed unnecessary [13, 30, 33, 44]. Some researchers have not classified iliac vein stenosis of 50% as an anatomical indication for IVS. Rizvi et al. [40] indicated that stenting should only be considered when residual stenosis after PTA remains at 50%. In contrast, Taha et al. [46] argue that a diameter stenosis of 50%, extensive intraluminal fibrosis, or a residual stenosis of up to 30% post-DVT with venous collaterals should all warrant stenting.

Maintaining continuous patency of the iliac vein stent is critical for achieving optimal postoperative outcomes. This review aimed to elucidate the favorable post-IVS CPPs. Through rigorous statistical analyses, several models with specific ES values were identified. The analysis yielded three key findings. First, the study by Hügel et al. [43], included in this review, reported a postoperative 2-year CPP of 84.3% for CIVO patients, with the 5-year CPP remaining high at 82.4%. Other studies reported that the postoperative 5-year CPP for ATIVO patients exceeds 90% [38, 42]. Additionally, meta-analysis revealed that the postoperative 2-year CPP for NIVCS patients was as high as 98.4%, indicating sustained high CPPs over time. Second, the post-IVS 6-month CPPs for PIVTS and ATIVO patients were 87.5% and 94.8%, respectively, both significantly lower than the 99.6% observed for NIVCS patients without thrombosis. The estimated 2-year CPP for CIVO patients was also markedly lower than that of NIVCS patients (71.0% vs 98.4%). These results showing higher postoperative CPPs of NIVCSs are consistent with the findings reported in multiple previous studies on different IVO populations [13, 29, 39, 44]. The above indicates that the presence of thrombus tissue is likely to have a significant negative impact on the patency of iliac vein stents. In depth, we believe that this may be related to the obstruction of the stent inflow and outflow caused by thrombosis [58, 59]. Unfortunately, in order to fully cover the thrombus in the inflow and/or outflow, the stent may need to be extended below the inguinal ligament and/or into the inferior vena cava, which will also affect the patency of the stent and increase the risk of contralateral DVT [11, 58]. Third, the estimated 6mo post-IVS CPP of ATIVOs seemed to be better than that of PIVTS (94.8% vs 87.5%). And Robertson et al.’s study [44], which analyzed the two groups of population separately, also reported a similar result (86.2% vs 84.2%). Most researchers believed that for ATIVOs, one of the prerequisites for implementing IVS is to use various thrombolysis modalities to almost completely remove the thrombus on the affected side [12, 15, 27, 31, 35]. This further confirms the importance of unobstructed inflow and outflow in maintaining stent patency.

In addition to the CPP, this review summarizes additional efficacy outcomes of IVS. Most of the studies included in this review indicated significant improvements in chronic symptoms—such as ulcers, edema, pain, and decreased QoL—for the majority of CIVOs. Moini et al. [13] reported a complete ulcer healing rate of 31%–42% at six months post-IVS for CIVOs. However, studies with longer follow-up periods (at least 17 months) reported significantly higher healing rates, with some reaching up to 100% [34, 41, 46]. This suggests that while IVS is effective for ulcer treatment, healing requires time. Additionally, numerous studies have confirmed the effectiveness of IVS in reducing edema and pain, as well as improving overall QoL [13, 16, 28, 30, 34, 41, 45, 46]. Therefore, we posit that the proven long-term patency of stents enables IVS to alleviate the symptoms of IVOs over an extended period and potentially prevent the progression of CVD.

Attention must also be given to the safety of IVS. The most frequently reported adverse event following IVS is back pain, with an incidence rate of up to 66% [37]. Despite this high incidence, studies indicate that the severity is generally mild and tends to resolve spontaneously, eliminating the need for special treatment [27, 29, 36, 37]. Some researchers have attributed this phenomenon to the larger stent diameter and the balloon angioplasty (PTA) performed prior to stent implantation [60, 61]. However, Snow et al. [37] argued that stent diameter and length are not predictive of back pain. The risk of complications associated with the stent as a foreign body has been a consistent concern. The most common complications reported include thrombosis, migration, fracture, and collapse, yet their incidences remain very low, often approaching 0%, regardless of the type of IVO [16, 33, 41, 46]. This low incidence is crucial for maintaining high stent patency. While two studies reported a 0% incidence of ipsilateral DVT following IVS for CIVOs, Kim et al. reported an 8.5% incidence of ipsilateral DVT for patients with ATIVOs at an average follow-up of 14 months postoperatively [16, 31, 41]. Kim et al. [31] suggested that ipsilateral DVT recurrence is linked to retained inferior vena cava filters and stent thrombosis. This indicates that in cases of ATIVOs, IVS may indirectly contribute to DVT recurrence in the ipsilateral lower limb through stent thrombosis. However, since the recurrence of DVT is influenced by numerous risk factors, further studies are required to determine whether IVS is an independent risk factor for ipsilateral lower limb DVT recurrence in ATIVOs. The incidence of contralateral DVT has been reported at 9%–11% within two to three years post-IVS [11, 15]. The excessive extension of the stent into the inferior vena cava, leading to compromised blood flow in the contralateral limb, is widely believed to correlate with contralateral DVT [11, 15, 31], and Kim et al. [31] also associated contralateral DVT with stent thrombosis. PTS is one of the primary long-term complications of ATIVOs. A controlled study by Ming et al. [12] indicated that IVS serves as an independent preventive factor for PTS in ATIVOs (Cox regression, odds ratio = 0.541, *P* ═ 0.012). Additionally, two other studies reported low PTS incidences (2.2% and 11.5%, respectively) following IVS [27, 35]. These rates represent cumulative outcomes over two years and are significantly lower than the over 40% PTS incidences reported within one year for proximal DVT patients receiving only anticoagulants or anticoagulants combined with thrombus removal procedures [62]. The mechanism by which IVS reduces PTS risk is believed to involve the restoration of iliac vein patency, thereby enhancing venous blood flow velocity in the lower limb [12, 63, 64]. Overall, this review suggests that IVS demonstrates a high level of safety across various IVOs.

It is important to acknowledge certain limitations within this study. First, the units used to calculate outcome incidences were inconsistent across the included studies. For instance, some studies defined the primary patency rate as the number of patients achieving primary patency divided by the total number of patients, while others substituted “patient” with “limb” in their calculations. This inconsistency may introduce error in pooled analyses. Second, patient selection criteria for those undergoing IVS varied among studies. Some studies positioned IVS as the initial preferred therapy for patients with confirmed IVO lesions, while others considered it an alternative following failed PTA. This selection bias could increase heterogeneity among studies. Third, the sample sizes for pooled analyses were limited, as there are few studies on IVS outcomes. To minimize heterogeneity, different IVO types and follow-up durations were distinguished, which further reduced sample sizes and consequently decreased statistical power. Finally, due to small sample sizes, tests for publication bias may have led to small sample effects, further diminishing statistical power.

While systematic reviews have summarized IVS outcomes, this study enhances understanding of its efficacy and safety by differentiating among various types of IVO patients and follow-up durations. Nevertheless, the number of relevant prospective controlled studies remains limited, and we anticipate the publication of additional research in this area.

## Conclusion

IVS effectively maintains long-term primary patency, improves clinical manifestations, and enhances QoL for patients with various IVOs. Moreover, IVS is deemed safe, warranting its important role in the treatment of IVOs.

## Supplemental data

Supplemental data are available at the following link: https://www.bjbms.org/ojs/index.php/bjbms/article/view/12777/4022.

## References

[1] May R, Thurner J (1957). The cause of the predominantly sinistral occurrence of thrombosis of the pelvic veins. Angiology.

[2] Cockett FB, Thomas ML (1965). The iliac compression syndrome. Br J Surg.

[3] De Maeseneer MG, Kakkos SK, Aherne T, Baekgaard N, Black S, Blomgren L (2022). Editor’s choice—European society for vascular surgery (ESVS) 2022 clinical practice guidelines on the management of chronic venous disease of the lower limbs. Eur J Vasc Endovasc Surg.

[4] Heller T, Teichert C, Hafer J, Weber MA, Kröger JC, Meinel FG (2019). Prevalence of May-Thurner syndrome in patients with deep vein thrombosis at a large medical referral center. Rofo.

[5] Cui YF, Fu YF, Liu HT, Hao X (2016). Combined catheter-directed thrombolysis and iliac vein recanalization for iliac vein compression syndrome with secondary acute deep vein thrombosis: efficacy and long-term outcome. Int Angiol.

[6] Yin M, Shi H, Ye K, Lu X, Li W, Huang X (2015). Clinical assessment of endovascular stenting compared with compression therapy alone in post-thrombotic patients with iliofemoral obstruction. Eur J Vasc Endovasc Surg.

[7] Blanch Alerany M, Izquierdo Lamoca LM, Ramirez Ortega M, Lago Rivas I, Zotta Desboeufs R, Stefanov Kiuri S (2014). Endovascular treatment of iliofemoral chronic post-thrombotic venous flow obstruction. J Vasc Surg Venous Lymphat Disord.

[8] de Wolf FMA, Arnoldussen CW, Grommes J, Hsien SG, Nelemans PJ, de Haan MW (2013). Minimally invasive treatment of chronic iliofemoral venous occlusive disease. J Vasc Surg Venous Lymphat Disord.

[9] Roy M, Sasson M, Rosales-Velderrain A, Moon S, Grove M, King T (2017). Pharmacomechanical thrombolysis for deep vein thrombosis in May-Thurner syndrome. Innovations (Phila).

[10] Jeon UB, Chung JW, Jae HJ, Kim HC, Kim SJ, Ha J (2010). May-Thurner syndrome complicated by acute iliofemoral vein thrombosis: helical CT venography for evaluation of long-term stent patency and changes in the iliac vein. AJR Am J Roentgenol.

[11] Le TB, Lee TK, Park KM, Jeon YS, Hong KC, Cho SG (2018). Contralateral deep vein thrombosis after iliac vein stent placement in patients with May-Thurner syndrome. J Vasc Interv Radiol.

[12] Ming ZB, Li WD, Yuan RF, Li XQ, Ding WB (2017). Efficacy of catheter directed thrombolysis and stent implantation on iliofemoral vein thrombosis caused by iliac vein compression. J Thromb Thrombolysis.

[13] Moini M, Zafarghandi MR, Taghavi M, Salimi J, Tadayon B, Mohammad Sadat SA (2020). Venoplasty and stenting in post-thrombotic syndrome and non-thrombotic iliac vein lesion. Minim Invasive Ther Allied Technol.

[14] Kwak HS, Han YM, Lee YS, Jin GY, Chung GH (2005). Stents in common iliac vein obstruction with acute ipsilateral deep venous thrombosis: early and late results. J Vasc Interv Radiol..

[15] Kim MS, Park HS, Hong HP, Hyun D, Cho SK, Park KB (2022). Risk factors for stent occlusion after catheter-directed thrombolysis and iliac vein stenting in the treatment of May-Thurner syndrome with iliofemoral deep vein thrombosis: a retrospective cohort study. Quant Imaging Med Surg.

[16] Tang TY, Yap CJQ, Chan SL, Soon SXY, Lim MHH, Tan JWH (2022). Midterm outcomes (2 years) using the Venovo™ and Sinus Obliquus™ venous stents in the treatment of non-thrombotic and post-thrombotic iliac vein lesions—Results from a multi-centre Asian cohort. Phlebology.

[17] Vasquez MA, Munschauer CE (2012). Revised venous clinical severity score: a facile measurement of outcomes in venous disease. Phlebology.

[18] Munn Z, Moola S, Lisy K, Riitano D, Tufanaru C (2015). Methodological guidance for systematic reviews of observational epidemiological studies reporting prevalence and cumulative incidence data. Int J Evid Based Healthc.

[19] Rostom A, Dubé C, Cranney A, Saloojee N, Sy R, Garritty C, et al.

[20] Nyaga VN, Arbyn M, Aerts M (2014). Metaprop: a Stata command to perform meta-analysis of binomial data. Arch Public Health.

[21] Mosteller F, Youtz C (1961). Tables of the Freeman-Tukey transformations for the binomial and Poisson distributions. Biometrika.

[22] Ried K (2006). Interpreting and understanding meta-analysis graphs: a practical guide. Aust Fam Phys.

[23] Thakkinstian A, McElduff P, D’Este C, Duffy D, Attia J (2005). A method for meta-analysis of molecular association studies. Stat Med.

[24] Whitehead A, Whitehead J (1991). A general parametric approach to the meta-analysis of randomized clinical trials. Stat Med.

[25] Egger M, Davey Smith G, Schneider M, Minder C (1997). Bias in meta-analysis detected by a simple, graphical test. BMJ.

[26] Biljana M, Jelena M, Branislav J, Milorad R (1999). Bias in meta-analysis and funnel plot asymmetry. Stud Health Technol Inform.

[27] Xue GH, Huang XZ, Ye M, Liang W, Zhang H, Zhang JW (2014). Catheter-directed thrombolysis and stenting in the treatment of iliac vein compression syndrome with acute iliofemoral deep vein thrombosis: outcome and follow-up. Ann Vasc Surg.

[28] Raju S, Owen S Jr, Neglen P (2002). The clinical impact of iliac venous stents in the management of chronic venous insufficiency. J Vasc Surg.

[29] Jiang L, Zhuang H, Song T, Li XQ (2024). Clinical outcomes at 3 years after stenting for thrombotic and non-thrombotic iliac vein compression syndrome patients. Clin Appl Thromb Hemost.

[30] Lim MNHH, Damodharan K, Chan SL, Toh MR, Yap CJQ, Chong TT (2020). Endovascular deep vein stenting of symptomatic post-thrombotic and non-thrombotic iliac vein stenotic lesions: a multicentre cohort experience from Singapore. Ann Acad Med Singap.

[31] Kim KY, Hwang HP, Han YM (2020). Factors affecting recurrent deep vein thrombosis after pharmacomechanical thrombolysis and left iliac vein stent placement in patients with iliac vein compression syndrome. J Vasc Interv Radiol.

[32] Satwah I, Sulakvelidze L, Tran M, Lakhanpal S, Kennedy R, Lakhanpal G (2022). Iliac vein stenting is safe when performed in an office based laboratory setting. J Vasc Surg Venous Lymphat Disord.

[33] Alsheekh A, Hingorani A, Ferm S, Kibrik P, Aurshina A, Marks N (2017). Is there an effect of race/ethnicity on early complications of iliac vein stenting?. Vascular.

[34] Ye K, Lu X, Li W, Huang Y, Huang X, Lu M (2012). Long-term outcomes of stent placement for symptomatic nonthrombotic iliac vein compression lesions in chronic venous disease. J Vasc Interv Radiol.

[35] Jiang C, Zhao Y, Wang X, Liu H, Tan TW, Li F (2020). Midterm outcome of pharmacomechanical catheter-directed thrombolysis combined with stenting for treatment of iliac vein compression syndrome with acute iliofemoral deep venous thrombosis. J Vasc Surg Venous Lymphat Disord.

[36] Raju S, Ward M Jr, Kirk O (2014). A modification of iliac vein stent technique. Ann Vasc Surg.

[37] Snow C, Pappas S, Sulakvelidze L, Kennedy R, Lakhanpal S, Pappas PJ (2023). Nitinol stents placed in iliac veins are not associated with prolonged back pain. Phlebology.

[38] Dasari M, Avgerinos E, Raju S, Tahara R, Chaer RA (2017). Outcomes of iliac vein stents after pregnancy. J Vasc Surg Venous Lymphat Disord.

[39] Abdul-Haqq R, Novak Z, Pearce BJ, Matthews TC, Patterson MA, Jordan WD Jr (2017). Routine extended follow-up surveillance of iliac vein stents for iliocaval venous obstruction may not be warranted. J Vasc Surg Venous Lymphat Disord.

[40] Rizvi SA, Ascher E, Hingorani A, Marks N (2018). Stent patency in patients with advanced chronic venous disease and nonthrombotic iliac vein lesions. J Vasc Surg Venous Lymphat Disord.

[41] Lichtenberg MKW, Stahlhoff WF, Stahlhoff S, Özkapi A, Breuckmann F, de Graaf R (2021). Venovo venous stent for treatment of non-thrombotic or post-thrombotic iliac vein lesions—Long-term efficacy and safety results from the Arnsberg venous registry. Vasa.

[42] Foegh P, Strandberg C, Joergensen S, Myschetzky PS, Klitfod L, Just S (2023). Long-term integrity of 53 iliac vein stents after catheter-directed thrombolysis. Acta Radiol.

[43] Hügel U, Khatami F, Muka T, Koeckerling D, Schindewolf M, Bernhard SM (2023). Criteria to predict midterm outcome after stenting of chronic iliac vein obstructions (PROMISE trial). J Vasc Surg Venous Lymphat Disord.

[44] Robertson B, Shapiro J, Muck A, Fellner AN, Recht M, Kulwicki A (2023). Venous stent patency is independent of total stented length in nonthrombotic iliac vein and post-thrombotic venous stenoses. J Vasc Surg Venous Lymphat Disord.

[45] Cooke PV, Bai H, Cho LD, Kang Y, Kim J, Dionne E (2022). Compression stocking compliance does not impact reintervention or symptom change after iliac vein stenting in patients with moderate to severe lower extremity edema. Ann Vasc Surg.

[46] Taha MAH, Hassan HA, Badawy AEH, Bahgat A.H, Thabet BAH (2020). Deep venous stenting in chronic iliac venous obstructive lesions: a single-center experience. Egypt J Surg.

[47] Beebe HG, Bergan JJ, Bergqvist D, Eklof B, Eriksson I, Goldman MP (1996). Classification and grading of chronic venous disease in the lower limbs. A consensus statement. Eur J Vasc Endovasc Surg.

[48] Kibbe MR, Ujiki M, Goodwin AL, Eskandari M, Yao J, Matsumura J (2004). Iliac vein compression in an asymptomatic patient population. J Vasc Surg.

[49] Chen D, Chen F, Li MF, Huang JG, Tang XH, Zhou WM (2018). Left iliac vein compression is not associated with infrainguinal deep venous thrombosis but is associated with iliac vein involvement. J Vasc Surg Venous Lymphat Disord.

[50] Kahn SR, Comerota AJ, Cushman M, Evans NS, Ginsberg JS, Goldenberg NA (2014). The postthrombotic syndrome: evidence-based prevention, diagnosis, and treatment strategies: a scientific statement from the American Heart Association. Circulation.

[51] Prandoni P, Lensing AW, Cogo A, Cuppini S, Villalta S, Carta M (1996). The long-term clinical course of acute deep venous thrombosis. Ann Intern Med.

[52] Villalta S, Bagatella P, Piccioli A, Lensing A, Prins M, Prandoni P (1994). Assessment of validity and reproducibility of a clinical scale for the post-thrombotic syndrome (abstract). Haemostasis.

[53] Delis KT, Bountouroglou D, Mansfield AO (2004). Venous claudication in iliofemoral thrombosis: long-term effects on venous hemodynamics, clinical status, and quality of life. Ann Surg.

[54] Eraslan BZ, Kodalak Cengiz S, İçmeli OS, Beyhan Sagmen S, Şener Cömert S (2024). Red cell distribution width to albumin ratio and mortality in acute pulmonary thromboembolism. Biomol Biomed..

[55] Wu MK, Luo XY, Zhang FX (2016). Incidence and risk factors of deep venous thrombosis in asymptomatic iliac vein compression: a prospective cohort study. Chin Med J (Engl).

[56] Razavi M, Lichtenberg M, Desai K, Dexter D, Soukas P, Shammas N (2025). The VIVID trial 12-month outcomes of the venous stent for the iliofemoral vein using the Duo venous stent system. J Vasc Surg Venous Lymphat Disord.

[57] Liu G, Qin J, Cui C, Ye K, Shi H, Liu X (2018). Comparison of direct iliofemoral stenting following angiojet rheolytic thrombectomy vs staged stenting after angiojet rheolytic thrombectomy plus catheter-directed thrombolysis in patients with acute deep vein thrombosis. J Endovasc Ther.

[58] Grøtta O, Enden T, Sandbæk G, Gjerdalen GF, Slagsvold CE, Bay D (2017). Patency and clinical outcome after stent placement for chronic obstruction of the inferior vena cava. Eur J Vasc Endovasc Surg.

[59] Neglén P, Raju S (2004). In-stent recurrent stenosis in stents placed in the lower extremity venous outflow tract. J Vasc Surg.

[60] Qiu P, Zha B, Xu A, Wang W, Zhan Y, Zhu X (2019). Systematic review and meta-analysis of iliofemoral stenting for post-thrombotic syndrome. Eur J Vasc Endovasc Surg.

[61] Rathore A, Gloviczki P, Bjarnason H (2016). Open surgical removal of iliac vein Wallstents with excision of pseudointima obstructing the contralateral iliac vein. J Vasc Surg Venous Lymphat Disord.

[62] Enden T, Haig Y, Kløw NE, Slagsvold CE, Sandvik L, Ghanima W (2012). Long-term outcome after additional catheter-directed thrombolysis versus standard treatment for acute iliofemoral deep vein thrombosis (the CaVenT study): a randomised controlled trial. Lancet.

[63] Badesha AS, Black SA, Khan G, Harper AJ, Thulasidasan N, Doyle A (2024). A meta-analysis of the medium- to long-term outcomes in patients with chronic deep venous disease treated with dedicated venous stents. J Vasc Surg Venous Lymphat Disord.

[64] Jalaie H, Barbati ME, Piao L, Doganci S, Kucher N, Dumantepe M (2025). Prognostic value of a classification system for iliofemoral stenting in patients with chronic venous obstruction. Eur J Vasc Endovasc Surg.

